# Care of the Eyes

**Published:** 1859-10

**Authors:** 


### CARE OF THE EYES.

Prescott, the historian, in consequence of a disorder of the nerve of the eye, wrote every word of his "*historicals*" without pen or ink, as he could not see when the pen was out of ink, or from any other cause failed to make a mark. He used an agate stylus on carbonated paper, the lines and edges of the page being indicated by brass wires in a wooden frame.

Crawford, the sculptor, the habit of whose life had been to read in a reclining position, lost one eye, and soon died from the formation of a malignant cancerous tumor behind the ball, which pushed it out on the cheek.

There are many affections of the eyes which are radically incurable. Persons of scrofulous constitutions, without any special local manifestation of it, often determine the disease to the eye by some erroneous habit or practice, and it remains there for life. It is useful, therefore, to know some of the causes which, by debilitating the eye, invite disease to it, or render it incapable of resisting adverse influences.

Avoid reading by candle or any other artificial light.

Reading by twilight ought never to be indulged in. A safe rule is—never read after sun-down, or before sun-rise.

Do not allow yourself to read a moment in any reclining position, whether in bed or on a sofa.

The practice of reading while on horseback, or in any vehicle in motion by wheels, is most pernicious.

Reading on steam or sail vessels should not be largely indulged in, because the slightest motion of the page on your body alters the focal point, and requires a painful, straining effort to readjust it.

Never attempt to look at the sun while shining unless through a colored glass of some kind: even a very bright moon should not be long gazed at.

The glare of the sun on water is very injurious to the sight.

A sudden change between bright light and darkness is always pernicious.

In looking at minute objects, relieve the eyes frequently by turning them to something in the distance.

Let the light, whether natural or artificial, fall on the page from behind, a little to one side.

Every parent should peremptorily forbid all sewing by candle or gas light, especially of dark materials.

If the eyes are matted together after sleeping, the most instantaneous and agreeable solvent in nature is the application of the saliva with the finger before opening the eye. Never pick it off with the finger nail, but wash it off with the ball of the fingers in quite warm water.

Never bathe or open the eyes in cold water. It is always safest, best, and most agreeable, to use warm water for that purpose, over seventy degrees.

